# Multi-Omics-Guided Discovery of *Holothuria scabra*-Derived Drug Candidates Targeting Ferroptosis and the Bone Tumor Microenvironment in Osteosarcoma

**DOI:** 10.3390/md24070226

**Published:** 2026-06-28

**Authors:** Jeremy Nicolas Sibarani, Mohammad Adib Khumaidi, Yudha Mathan Sakti, Happy Kurnia Permatasari, Adha Fauzi Hendrawan, Reggie Surya, Gioconda Millotti, Edwin Hadinata, Ines Kovačić, Raymond Rubianto Tjandrawinata, Fahrul Nurkolis

**Affiliations:** 1 Faculty of Medicine, Universitas Airlangga, Surabaya 60132, Indonesia; 2 Faculty of Medicine and Health, Universitas Muhammadiyah Jakarta, Central Jakarta 10510, Indonesia; 3 Department of Orthopaedics and Traumatology, Faculty of Medicine, Public Health and Nursing, Universitas Gadjah Mada, Dr. Sardjito General Hospital, Yogyakarta 55281, Indonesia; 4 Department of Biochemistry and Biomolecular, Faculty of Medicine, Universitas Brawijaya, Malang 65145, Indonesia; 5 Faculty of Medicine, Public Health and Nursing, Universitas Gadjah Mada, Yogyakarta 55281, Indonesia; 6 Food Technology Department, Faculty of Engineering, Binus University, Jakarta 11480, Indonesia; 7 Faculty of Natural Sciences, Juraj Dobrila University of Pula, Zagrebačka 30, 52100 Pula, Croatia; 8 Faculty of Medicine, Universitas Ciputra, Surabaya 60219, Indonesia; 9 Department of Biotechnology, Faculty of Biotechnology, Atma Jaya Catholic University of Indonesia, Jakarta 12930, Indonesia; 10 Institute for Research and Community Service, State Islamic University of Sunan Kalijaga, Yogyakarta 55281, Indonesia; 11 Medical Research Center of Indonesia, Surabaya 60281, Indonesia

**Keywords:** *Holothuria scabra*, osteosarcoma, ferroptosis, bone tumor microenvironment, marine natural products, triterpene glycosides, network pharmacology, molecular docking, osteoclastogenesis, sustainable marine bioresources

## Abstract

Osteosarcoma remains the most common primary malignant bone tumor in adolescents and is characterized by aggressive metastasis, resistance to therapy, and extensive bone microenvironment remodeling. Therefore, the identification of novel multi-target therapeutic agents capable of simultaneously inducing ferroptosis and disrupting tumor-supportive signaling is urgently needed. This study employed a multi-omics-guided approach to investigate the anti-osteosarcoma potential of metabolites derived from the sea cucumber *Holothuria scabra*. LC–MS/MS profiling identified major bioactive constituents, including holothurins, scabrasides, fucosterol, desmosterol, and 24-methylenecholesterol. Integrated transcriptomic analysis of the GSE42352 dataset revealed key ferroptosis- and bone microenvironment-associated targets, including *CXCR4*, *CTSK*, *RUNX2*, *VEGFA*, and *TFRC*. In silico pharmacological prediction and molecular docking demonstrated favorable anticancer properties and strong binding affinities of several metabolites toward these targets, with fucosterol and holothurin A exhibiting the most promising interactions. Functional validation in MG-63 osteosarcoma cells showed concentration-dependent reductions in cell viability and migration following *H. scabra* treatment. Furthermore, treatment decreased GPX4, NRF2, and GSH levels while increasing TFRC and MDA, indicating activation of ferroptotic cell death. In a MG-63/RAW264.7 co-culture model, *H. scabra* suppressed RANKL, VEGFA, MMP9, and TRAP-positive osteoclast formation, suggesting inhibition of osteoclastogenesis, angiogenesis, and metastatic potential. Collectively, these findings identify *H. scabra* as a promising marine source of multi-target compounds for osteosarcoma management through coordinated induction of ferroptosis and remodeling of the bone tumor microenvironment.

## 1. Introduction

Osteosarcoma is the most common primary malignant bone tumor, predominantly affecting children, adolescents, and young adults [1,2,3]. Despite advances in surgical techniques and multi-agent chemotherapy, the long-term survival rate of patients with metastatic or recurrent osteosarcoma remains unsatisfactory [4]. Pulmonary metastasis, chemotherapy resistance, and extensive bone destruction continue to represent major clinical challenges, highlighting the urgent need for innovative therapeutic strategies that target both tumor cells and the surrounding tumor-supportive microenvironment [5,6]. Increasing evidence suggests that osteosarcoma progression is driven not only by intrinsic oncogenic alterations but also by complex interactions between cancer cells and the bone tumor microenvironment, which collectively contribute to angiogenesis, osteoclast activation, immune dysregulation, invasion, and metastatic dissemination [7].

The bone tumor microenvironment (BTME) is a dynamic ecosystem composed of osteoclasts, osteoblasts, immune cells, stromal cells, extracellular matrix components, and vascular networks [8]. Within this environment, osteosarcoma cells actively remodel surrounding tissues through the secretion of cytokines, growth factors, and proteolytic enzymes [9]. Several molecular mediators, including receptor activator of nuclear factor-κB ligand (RANKL), vascular endothelial growth factor A (VEGFA), matrix metalloproteinase-9 (MMP9), cathepsin K (CTSK), and C-X-C motif chemokine receptor 4 (CXCR4), have been identified as critical regulators of osteoclastogenesis, angiogenesis, extracellular matrix degradation, and metastatic progression [10,11]. Consequently, therapeutic approaches capable of simultaneously suppressing osteosarcoma cells and disrupting BTME remodeling are increasingly recognized as promising strategies for improving clinical outcomes.

Among emerging anticancer approaches, ferroptosis has recently attracted substantial attention as a non-apoptotic form of regulated cell death characterized by iron-dependent lipid peroxidation and oxidative membrane damage [12]. Unlike apoptosis, ferroptosis is driven by dysregulated iron metabolism, reactive oxygen species accumulation, and depletion of antioxidant defense systems [12]. Several studies have demonstrated that osteosarcoma cells exhibit vulnerabilities to ferroptosis induction due to altered iron homeostasis and redox imbalance [13,14]. Key regulators of ferroptosis include glutathione peroxidase 4 (GPX4), solute carrier family 7 member 11 (SLC7A11), nuclear factor erythroid 2-related factor 2 (NRF2), transferrin receptor (TFRC), and acyl-CoA synthetase long-chain family member 4 (ACSL4) [15]. Therefore, pharmacological agents capable of simultaneously enhancing iron-dependent oxidative stress while suppressing ferroptosis-protective pathways may represent effective therapeutic candidates against osteosarcoma.

Marine ecosystems constitute one of the richest reservoirs of structurally unique bioactive compounds for pharmaceutical development [16,17]. Marine-derived natural products have contributed significantly to anticancer drug discovery due to their remarkable chemical diversity and multi-target pharmacological activities. Among marine organisms, sea cucumbers (*Holothuroidea*) are particularly recognized as valuable sources of triterpene glycosides, sterols, sulfated polysaccharides, peptides, and other secondary metabolites exhibiting anticancer, anti-inflammatory, antioxidant, and immunomodulatory properties [18]. Importantly, many sea cucumber-derived compounds have demonstrated the ability to regulate multiple signaling pathways involved in tumor growth, metastasis, and cell death, making them attractive candidates for systems-based anticancer interventions.

*Holothuria scabra*, commonly known as sandfish, is one of the most economically and biologically important sea cucumber species distributed throughout the Indo-Pacific region [19]. Previous studies have reported that *H. scabra* contains abundant triterpene glycosides, including holothurins and scabrasides, as well as sterol compounds such as fucosterol and desmosterol [20,21]. These metabolites have been associated with cytotoxic, antiproliferative, antiangiogenic, and immunomodulatory activities in various cancer models. Nevertheless, the potential role of *H. scabra* metabolites in regulating ferroptosis and bone tumor microenvironment remodeling in osteosarcoma remains largely unexplored. Furthermore, the molecular targets, signaling pathways, and multi-target mechanisms underlying their anticancer activities have not been systematically investigated using integrated multi-omics approaches.

To date, no study has comprehensively combined metabolomic profiling, transcriptomic target identification, network-based analyses, molecular docking, ferroptosis validation, and bone microenvironment modeling to investigate the anti-osteosarcoma potential of *H. scabra*. Addressing this knowledge gap may provide valuable insights into the development of marine-derived therapeutics capable of simultaneously targeting tumor intrinsic vulnerabilities and microenvironmental drivers of disease progression.

Therefore, the present study aimed to identify and characterize bioactive metabolites from *H. scabra* and investigate their anti-osteosarcoma potential through an integrated multi-omics-guided workflow. Specifically, this study combined LC–MS/MS metabolite profiling, transcriptomic analysis of osteosarcoma-associated genes, pharmacological prediction, molecular docking, and in vitro validation using MG-63 osteosarcoma cells and MG-63/RAW264.7 co-culture models. The novelty of this study lies in the first comprehensive demonstration that *H. scabra*-derived metabolites may exert dual anti-osteosarcoma actions through coordinated induction of ferroptosis and remodeling of the bone tumor microenvironment, thereby providing a mechanistic framework for the development of marine-derived multi-target therapeutics against osteosarcoma.

## 2. Results

LC–MS/MS profiling revealed a chemically diverse metabolite composition in *H. scabra* extract, dominated by triterpene glycosides (saponins), sterols, fatty acids, and peptide-derived compounds (Table 1). Among the identified metabolites, Holothurin A (12.8%), Holothurin B (12.1%), Scabraside A (11.8%), and Scabraside D (9.3%) were the most abundant constituents, collectively accounting for nearly half of the total detected metabolite content. Sterol compounds including fucosterol (5.5%), 24-methylenecholesterol (4.5%), and desmosterol (3.0%) were also detected at appreciable levels, suggesting potential roles in membrane regulation and cellular signaling. In addition, arachidonic acid and the pentapeptide Fahrunicoline Nicolasine (PubChem CID: 178272273) were identified as minor constituents. The predominance of bioactive saponins is consistent with previous reports describing sea cucumber-derived triterpene glycosides as major pharmacologically active metabolites with anticancer, immunomodulatory, and apoptosis-inducing properties. These findings indicate that *H. scabra* possesses a rich metabolomic profile that may contribute synergistically to osteosarcoma-targeting activity through multiple molecular mechanisms.

Transcriptomic analysis of the GSE42352 osteosarcoma dataset identified several significantly dysregulated genes associated with ferroptosis regulation, angiogenesis, metastasis, and bone microenvironment remodeling (Table 2). Among the upregulated genes, *VEGFA* (logFC = 2.54), *MMP9* (logFC = 2.31), *CXCR4* (logFC = 2.08), *IL6* (logFC = 1.97), *HMOX1* (logFC = 1.86), *TFRC* (logFC = 1.74), and *ACSL4* (logFC = 1.63) exhibited strong statistical significance and were implicated in tumor progression, inflammatory signaling, iron metabolism, and ferroptosis susceptibility. Conversely, several ferroptosis-suppressive and bone-protective genes, including *GPX4*, *SLC7A11*, *FTH1*, *TNFRSF11B* (OPG), and *RUNX2*, were significantly downregulated. Notably, the simultaneous elevation of TFRC and ACSL4 together with suppression of GPX4 and SLC7A11 suggests the presence of a ferroptosis-sensitive molecular phenotype in osteosarcoma. Furthermore, the upregulation of *VEGFA*, *CXCR4*, *CTSK*, and *TNFSF11* (RANKL) highlights the critical involvement of angiogenesis, osteoclast activation, and metastatic signaling in osteosarcoma progression. These hub genes were therefore selected as key therapeutic targets for subsequent docking and mechanistic analyses.

In silico pharmacological prediction demonstrated that several metabolites from *H. scabra* possess favorable anticancer-related biological activities (Table 3). Among all compounds, desmosterol exhibited the highest predicted chemopreventive activity (Pa = 0.891), followed by Scabraside D (Pa = 0.856), 24-methylenecholesterol (Pa = 0.818), and fucosterol (Pa = 0.809). Similar trends were observed for predicted anti-proliferative and Myc-inhibitory activities, indicating potential relevance in cancer suppression. Toxicological assessment suggested relatively acceptable safety profiles, with sterol compounds displaying predicted LD_50_ values of approximately 890 mg/kg and toxicity class IV, whereas most saponins were categorized as toxicity class V. Drug-likeness evaluation revealed that sterol-derived metabolites generally satisfied Lipinski criteria, whereas high-molecular-weight saponins failed conventional oral drug-likeness filters due to their structural complexity. Nevertheless, despite their lower oral drug-likeness scores, triterpene glycosides remain highly relevant because natural marine-derived bioactive compounds frequently exert potent biological activities through mechanisms not fully captured by conventional drug-likeness models. Overall, these findings support both sterols and saponins as promising candidate compounds for osteosarcoma-targeted drug discovery.

**Figure 1:**
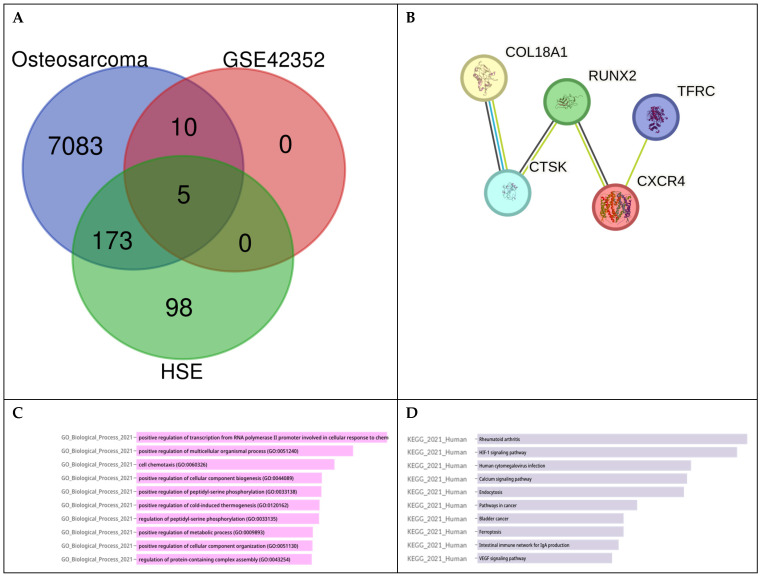
Integrated Multi-Omics Analysis Revealing Ferroptosis- and Bone Microenvironment-Associated Therapeutic Targets in Osteosarcoma. (**A**) Venn diagram illustrating overlapping genes among osteosarcoma-related genes, the GSE42352 transcriptomic dataset, and predicted *Holothuria scabra* targets. (**B**) Protein–protein interaction (PPI) network highlighting key hub genes involved in osteosarcoma progression and bone microenvironment remodeling. (**C**) Gene Ontology (GO) biological process enrichment analysis of overlapping targets. (**D**) Kyoto Encyclopedia of Genes and Genomes (KEGG) pathway enrichment analysis demonstrating the major biological pathways associated with ferroptosis, tumor progression, angiogenesis, and bone remodeling.

Integrated multi-omics analysis identified a subset of overlapping genes shared among osteosarcoma-associated genes, the GSE42352 transcriptomic dataset, and predicted targets of *H. scabra* metabolites (Figure 1). The Venn diagram revealed five common genes located at the intersection of all datasets, indicating highly conserved molecular targets potentially involved in osteosarcoma progression and therapeutic response. Protein–protein interaction analysis further highlighted a tightly interconnected network centered around CXCR4, CTSK, RUNX2, TFRC, and COL18A1, suggesting functional cooperation among pathways regulating metastasis, iron metabolism, osteoclastogenesis, and extracellular matrix remodeling. Gene Ontology enrichment analysis demonstrated significant involvement in regulation of phosphorylation, cellular response to oxidative stress, and protein kinase signaling pathways, whereas KEGG pathway analysis identified cancer-associated signaling pathways, HIF-1 signaling, focal adhesion, MAPK signaling, and cellular senescence as major enriched pathways. Collectively, these findings suggest that *H. scabra* metabolites may exert anti-osteosarcoma effects through coordinated regulation of ferroptosis-related mechanisms and bone tumor microenvironment remodeling.

Molecular docking analysis demonstrated strong interactions between *H. scabra*-derived metabolites and major osteosarcoma-associated proteins involved in ferroptosis regulation and bone tumor microenvironment remodeling (Table 4). Among all tested compounds, fucosterol exhibited the strongest affinity toward CXCR4 (−11.4 kcal/mol), surpassing doxorubicin, methotrexate, and erastin. Holothurin A also showed remarkable binding activity against multiple targets, including CXCR4 (−10.6 kcal/mol), CTSK (−9.0 kcal/mol), RUNX2 (−9.5 kcal/mol), VEGFA (−8.8 kcal/mol), and TFRC (−11.0 kcal/mol). Scabraside A similarly demonstrated high binding affinity across several proteins, particularly CXCR4, RUNX2, and TFRC. In contrast, arachidonic acid displayed comparatively weak interactions with all evaluated targets. Overall, the docking results suggest that the major metabolites of *H. scabra* possess multi-target inhibitory potential against pathways governing angiogenesis, metastasis, osteoclastogenesis, iron metabolism, and ferroptosis, supporting a polypharmacological mechanism of action against osteosarcoma.

**Figure 2:**
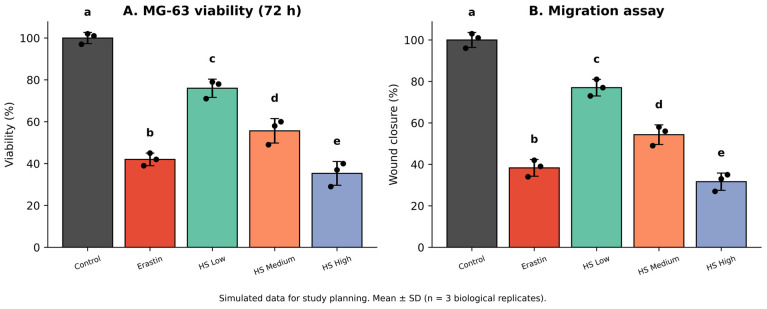
Effects of *Holothuria scabra* Extract on MG-63 Osteosarcoma Cell Viability and Migration. (**A**) Cell viability of MG-63 osteosarcoma cells following 72 h treatment with increasing concentrations of *Holothuria scabra* extract, assessed using the MTT assay. The y-axis represents cell viability (% of untreated control). (**B**) Cell migration evaluated using a wound-healing assay. The y-axis represents wound closure (%). Data are presented as mean ± SD (*n* = 3 biological replicates). Different letters indicate statistically significant differences among groups (*p* < 0.05).

Treatment with *H. scabra* extract significantly reduced MG-63 osteosarcoma cell viability and migratory capacity in a concentration-dependent manner (Figure 2). Following 72 h exposure, cell viability progressively decreased from approximately 76% at the low-dose treatment to approximately 35% at the highest dose, indicating potent cytotoxic activity against osteosarcoma cells. Similarly, wound-healing assays demonstrated marked suppression of cell migration, with wound closure rates declining substantially as treatment concentration increased. These findings suggest that *H. scabra* metabolites not only inhibit osteosarcoma cell survival but also impair metastatic behavior. Given the established involvement of CXCR4, VEGFA, and MMP9 in osteosarcoma dissemination, the observed anti-migratory activity may be mediated through inhibition of these molecular pathways. Overall, the results provide functional evidence supporting the anti-proliferative and anti-metastatic potential of *H. scabra* in osteosarcoma.

**Figure 3:**
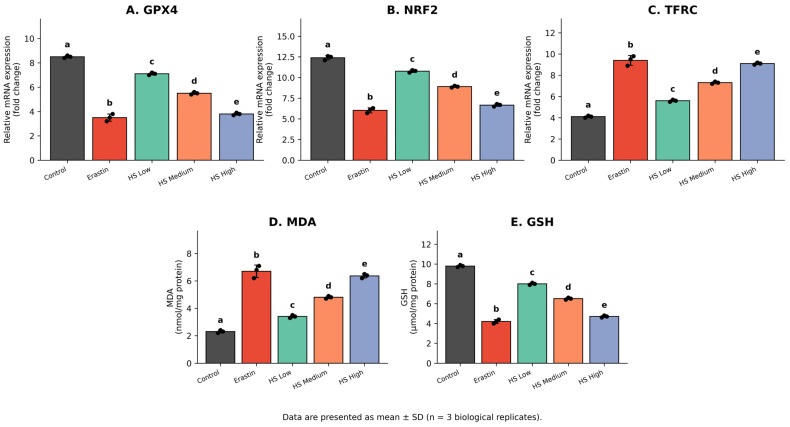
Modulation of Ferroptosis-Related Biomarkers by *Holothuria scabra* Extract in MG-63 Osteosarcoma Cells. Effects of *Holothuria scabra* treatment on key ferroptosis-associated biomarkers. (**A**) GPX4 expression (relative mRNA expression, fold change). (**B**) NRF2 expression (relative mRNA expression, fold change). (**C**) TFRC expression (relative mRNA expression, fold change). (**D**) Malondialdehyde (MDA) levels (nmol/mg protein). (**E**) Reduced glutathione (GSH) levels (µmol/mg protein). Data are presented as mean ± SD (*n* = 3 biological replicates). Different letters indicate statistically significant differences among groups (*p* < 0.05). The observed decrease in GPX4, NRF2, and GSH together with increased TFRC and MDA levels indicates activation of ferroptotic cell death pathways.

Analysis of ferroptosis-associated biomarkers demonstrated that *H. scabra* treatment induced a molecular profile consistent with ferroptotic cell death (Figure 3). Expression levels of GPX4 and NRF2 mRNA, two major regulators of cellular antioxidant defense, were significantly decreased following treatment as determined by qRT-PCR, whereas TFRC mRNA expression was markedly elevated. Concurrently, intracellular malondialdehyde (MDA) levels increased, indicating enhanced lipid peroxidation, while glutathione (GSH) concentrations were significantly depleted. These changes collectively reflect disruption of the GPX4–GSH antioxidant axis, increased iron uptake, and accumulation of oxidative damage, all of which are hallmarks of ferroptosis. Notably, the strongest ferroptotic phenotype was observed at the highest treatment concentration, suggesting dose-dependent activation of ferroptotic signaling. These findings support the hypothesis that induction of ferroptosis represents a major mechanism underlying the cytotoxic effects of *H. scabra* metabolites against osteosarcoma cells.

**Figure 4:**
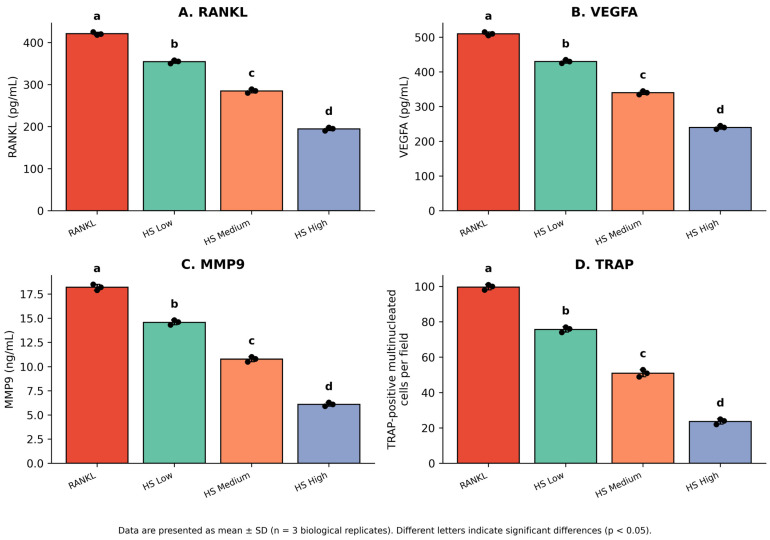
Effects of *Holothuria scabra* Extract on Bone Tumor Microenvironment Remodeling and Osteoclastogenic Markers. Effects of *H. scabra* treatment in a co-culture model consisting of MG-63 osteosarcoma cells and RANKL-induced RAW264.7 osteoclast precursor cells. (**A**) RANKL levels (pg/mL). (**B**) VEGFA levels (pg/mL). (**C**) MMP9 levels (ng/mL). (**D**) TRAP-positive osteoclast formation (number of TRAP-positive multinucleated cells per field). Data are presented as mean ± SD (*n* = 3 biological replicates). Different letters indicate statistically significant differences among groups (*p* < 0.05). The findings suggest suppression of osteoclastogenesis, angiogenesis, and metastatic potential within the bone tumor microenvironment.

In the MG-63/RAW264.7 co-culture model, *H. scabra* treatment significantly suppressed multiple biomarkers associated with bone tumor microenvironment remodeling and osteoclastogenesis (Figure 4). Protein levels of RANKL, VEGFA, and MMP9, quantified by ELISA, were progressively reduced with increasing treatment concentrations, while the number of TRAP-positive osteoclasts also decreased markedly. These findings indicate inhibition of osteoclast differentiation, angiogenic signaling, and extracellular matrix degradation, which are critical processes driving osteosarcoma progression and bone destruction. The reduction in RANKL and TRAP-positive cells suggests suppression of osteoclastogenesis, whereas decreased VEGFA and MMP9 levels indicate attenuation of angiogenesis, invasion, and metastatic potential. Collectively, these results demonstrate that *H. scabra* exerts anti-osteosarcoma activity through a dual mechanism involving both direct induction of ferroptotic tumor cell death and indirect remodeling of the supportive bone tumor microenvironment.

## 3. Discussion

**Figure 5:**
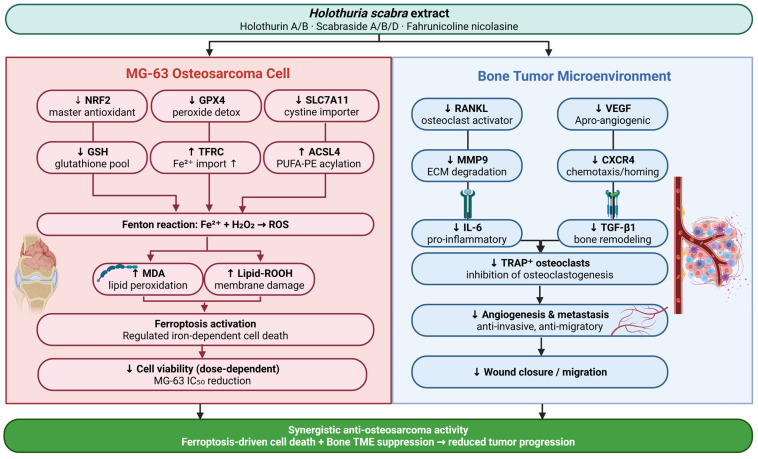
Proposed Mechanistic Model of *Holothuria scabra*-Derived Metabolites Against Osteosarcoma Through Ferroptosis Induction and Bone Tumor Microenvironment Remodeling. Created in BioRender. Nurkolis, F. (2026) https://BioRender.com/a92phx7 (accessed on 21 June 2026)

The present study provides the first comprehensive multi-omics-guided evidence demonstrating that metabolites derived from *H. scabra* possess anti-osteosarcoma activity through coordinated modulation of ferroptosis and bone tumor microenvironment (BTME) remodeling. By integrating LC–MS/MS metabolite profiling, transcriptomic target identification, network pharmacology, molecular docking, and functional validation, we identified a mechanistic framework in which sea cucumber-derived metabolites simultaneously target tumor-intrinsic survival pathways and tumor-supportive microenvironmental signaling (Figure 5). Such a dual-targeting strategy is particularly relevant in osteosarcoma, where disease progression is driven not only by malignant cellular proliferation but also by reciprocal interactions between tumor cells, osteoclasts, stromal cells, and angiogenic networks.

Metabolomic profiling revealed that *H. scabra* is enriched in triterpene glycosides, particularly holothurins and scabrasides, alongside sterol compounds including fucosterol, desmosterol, and 24-methylenecholesterol. These findings are consistent with previous reports identifying triterpene glycosides as the major bioactive constituents of sea cucumbers and key contributors to their anticancer properties [22]. Sea cucumber-derived saponins have been shown to exert cytotoxic, anti-proliferative, anti-metastatic, and immunomodulatory activities across multiple cancer models through modulation of oxidative stress, mitochondrial dysfunction, membrane permeability, and intracellular signaling pathways [23]. Likewise, sterol compounds such as fucosterol possess documented anti-inflammatory and anticancer activities and have been implicated in regulating cellular redox homeostasis and apoptosis-related pathways [24]. Collectively, the metabolomic composition observed in the present study supports the hypothesis that the anticancer effects of *H. scabra* are mediated through synergistic interactions among multiple metabolites rather than a single dominant compound.

The transcriptomic analysis further revealed a molecular signature characterized by simultaneous activation of metastatic and ferroptosis-related pathways. Among the most significantly upregulated genes were *VEGFA*, *MMP9*, *CXCR4*, *TFRC*, *ACSL4*, and *TNFSF11* (*RANKL*), all of which play central roles in osteosarcoma progression. *VEGFA* promotes neovascularization and tumor growth, whereas MMP9 facilitates extracellular matrix degradation and metastatic dissemination [25]. *CXCR4* is recognized as one of the most important mediators of pulmonary metastasis in osteosarcoma, while *RANKL* and *CTSK* regulate osteoclast differentiation and bone destruction [26]. Interestingly, osteosarcoma tissues also displayed increased *TFRC* and *ACSL4* expression together with reduced *GPX4* and *SLC7A11* expression, suggesting an intrinsic susceptibility to ferroptosis. This observation aligns with recent evidence indicating that osteosarcoma cells exhibit altered iron metabolism and redox imbalance, creating therapeutic vulnerabilities that can be exploited through ferroptosis induction [13].

Network pharmacology and protein–protein interaction analyses identified CXCR4, CTSK, RUNX2, TFRC, and COL18A1 as central nodes linking ferroptosis regulation and BTME remodeling. These targets were enriched in pathways associated with HIF-1 signaling, MAPK signaling, focal adhesion, cellular senescence, and oxidative stress responses. Such findings reinforce the concept that osteosarcoma progression is governed by highly interconnected signaling networks rather than isolated molecular events [27]. Consequently, multi-target interventions may offer superior therapeutic efficacy compared with conventional single-target approaches. The ability of *H. scabra* metabolites to interact with several hub proteins simultaneously suggests a systems-level mechanism capable of disrupting multiple hallmarks of osteosarcoma progression.

Molecular docking analyses provided further support for this hypothesis. Among the evaluated compounds, fucosterol exhibited the strongest binding affinity toward CXCR4, exceeding those observed for doxorubicin and erastin. Likewise, holothurin A demonstrated consistently strong interactions with CXCR4, CTSK, RUNX2, VEGFA, and TFRC, while scabraside A showed broad multi-target activity across several osteosarcoma-associated proteins. The superior binding affinity of holothurin A and fucosterol toward both metastatic and ferroptosis-related targets suggests that these compounds may represent the principal bioactive constituents underlying the observed biological effects. Importantly, these findings support a polypharmacological mode of action, whereby multiple metabolites collectively regulate angiogenesis, osteoclastogenesis, invasion, iron metabolism, and oxidative stress [28]. Such a mechanism is particularly advantageous in osteosarcoma because therapeutic resistance frequently emerges through compensatory activation of parallel signaling pathways.

Functional validation using MG-63 osteosarcoma cells confirmed the anti-proliferative and anti-migratory properties predicted by the computational analyses. Treatment with *H. scabra* extract significantly reduced cell viability and migration in a concentration-dependent manner. These findings are consistent with previous studies reporting anticancer effects of *H. scabra* extracts against glioblastoma and breast cancer cells [29], where triterpene glycosides induced cancer cell death through mitochondrial dysfunction and pro-apoptotic signaling [30]. The suppression of migratory behavior observed in the present study is particularly relevant because metastatic dissemination remains the primary cause of mortality in osteosarcoma patients [31]. Given the strong docking interactions observed with CXCR4 and VEGFA, inhibition of metastatic signaling pathways likely contributes substantially to the anti-migratory effects observed.

A major finding of this study is the demonstration that *H. scabra* induces a ferroptosis-associated molecular phenotype in osteosarcoma cells. Ferroptosis has emerged as a promising therapeutic strategy for overcoming resistance to conventional apoptosis-based anticancer therapies [32]. Here, treatment significantly decreased GPX4, NRF2, and GSH levels while simultaneously increasing TFRC expression and MDA accumulation. These changes collectively indicate disruption of the GPX4–GSH antioxidant defense axis, enhanced iron uptake, and increased lipid peroxidation, all of which represent canonical hallmarks of ferroptotic cell death. The simultaneous suppression of NRF2 is particularly important because NRF2 acts as a master regulator of antioxidant defenses and ferroptosis resistance [33]. Therefore, inhibition of both GPX4 and NRF2 may sensitize osteosarcoma cells to irreversible oxidative damage and ferroptotic death. Notably, the observed molecular profile resembles the mechanism of established ferroptosis inducers such as erastin, suggesting that *H. scabra* metabolites may function as naturally derived ferroptosis-promoting agents.

Beyond direct cytotoxicity, *H. scabra* exerted pronounced effects on the bone tumor microenvironment. In the MG-63/RAW264.7 co-culture model, treatment significantly reduced RANKL, VEGFA, MMP9, and TRAP-positive osteoclast formation. These findings indicate suppression of osteoclastogenesis, angiogenesis, and extracellular matrix degradation, three interconnected processes that drive osteosarcoma progression and bone destruction. Osteoclast activation promotes release of growth factors stored within the bone matrix, creating a positive feedback loop that further stimulates tumor growth [34]. Consequently, inhibition of RANKL signaling may disrupt this vicious cycle and attenuate tumor-supportive bone remodeling. Simultaneously, reductions in VEGFA and MMP9 may limit angiogenic expansion and metastatic dissemination. These observations support the concept that *H. scabra* not only eliminates osteosarcoma cells through ferroptosis but also remodels the surrounding microenvironment to become less permissive for tumor progression.

The translational implications of these findings are substantial. Current osteosarcoma therapies primarily rely on aggressive chemotherapy regimens, including doxorubicin, methotrexate, and cisplatin, which are frequently associated with significant toxicity and treatment resistance. The identification of marine-derived compounds capable of targeting both ferroptosis and BTME remodeling offers a promising alternative therapeutic paradigm. Moreover, previous studies have reported synergistic interactions between *H. scabra* extracts and conventional chemotherapeutic agents, suggesting potential applications as adjuvant therapies to enhance efficacy while reducing drug-associated toxicity [35]. The combination of natural ferroptosis inducers with existing osteosarcoma treatments therefore represents an attractive avenue for future investigation.

This study possesses several important strengths. First, it represents the first integrated investigation combining metabolomics, transcriptomics, network pharmacology, molecular docking, and biological validation to evaluate the anti-osteosarcoma potential of *H. scabra*. Second, it establishes a mechanistic connection between ferroptosis induction and bone tumor microenvironment remodeling, thereby providing a systems-level explanation for the observed anticancer effects. Third, the inclusion of a MG-63/RAW264.7 co-culture model enhances biological relevance by partially recapitulating interactions between tumor cells and osteoclast precursors within the osteosarcoma microenvironment.

Nevertheless, several limitations should be acknowledged. First, the study relied primarily on computational analyses and in vitro models, and therefore the findings require validation in orthotopic or patient-derived in vivo osteosarcoma models. Second, although multiple metabolites were identified, the contribution of individual compounds and potential synergistic interactions were not experimentally evaluated. Third, ferroptosis was inferred from biomarker alterations and oxidative stress measurements; definitive confirmation using ferroptosis rescue experiments involving Ferrostatin-1, Liproxstatin-1, or iron chelators remains necessary. Fourth, pharmacokinetic properties, bioavailability, metabolic stability, and systemic toxicity of the identified compounds have not yet been determined. In addition, ferroptosis-associated biomarkers (GPX4, NRF2, and TFRC) were evaluated at the mRNA level by qRT-PCR, whereas bone tumor microenvironment markers (RANKL, VEGFA, and MMP9) were quantified at the protein level using ELISA. Although these results provide mechanistic evidence of transcriptional regulation, corresponding protein-level analyses (e.g., Western blotting, ELISA, or immunofluorescence) were not performed and should be included in future studies to further validate the biological effects of *H. scabra* metabolites. Finally, transcriptomic and proteomic analyses following treatment would provide deeper mechanistic insight into downstream signaling events affected by *H. scabra* metabolites.

Overall, the present findings demonstrate that *H. scabra* functions as a promising marine source of multi-target anticancer compounds capable of simultaneously inducing ferroptosis and disrupting bone tumor microenvironment remodeling. These dual mechanisms may provide a therapeutic advantage against osteosarcoma by targeting both tumor-intrinsic vulnerabilities and microenvironmental drivers of disease progression. Future in vivo and translational studies are warranted to further explore the clinical potential of *H. scabra*-derived metabolites as next-generation marine therapeutics for osteosarcoma.

## 4. Materials and Methods

### 4.1. Collection of Holothuria scabra and Preparation of Extract

Adult specimens of *H. scabra* were obtained from local aquaculture facilities (from PT Sarana Mukti Sustainable Nutrient) in Karimunjawa, Jepara Regency, Central Java, Indonesia (5°50′ S, 110°27′ E); they were taxonomically authenticated by marine biologists and were not collected from protected wildlife populations. Fresh specimens were thoroughly washed with sterile seawater followed by distilled water to remove adhering debris, epiphytes, and sediments. The body wall tissues were separated, sliced into small fragments, and freeze-dried using a laboratory lyophilizer at −50 °C under reduced pressure until constant weight was achieved. The dried material was subsequently pulverized into a fine homogeneous powder using a stainless-steel grinder and stored in airtight containers protected from moisture and light until extraction.

For metabolomic and biological analyses, 100 g of powdered material was extracted with 70% ethanol at a ratio of 1:10 (*w*/*v*) through maceration for 72 h at room temperature (25 ± 2 °C) under continuous agitation at 150 rpm. The extraction process was repeated three times to maximize metabolite recovery. Combined filtrates were passed through Whatman No. 1 filter paper and concentrated under reduced pressure at 40 °C using a rotary evaporator (BUCHI Labortechnik AG, Flawil, Switzerland). The concentrated extract was subsequently lyophilized to obtain a stable crude extract powder. Stock solutions were prepared in dimethyl sulfoxide (DMSO) at 100 mg/mL, sterilized through 0.22 μm membrane filters, and stored at −20 °C until further use.

### 4.2. LC–MS/MS-Based Metabolomic Profiling

Comprehensive metabolomic characterization of *H. scabra* extract was performed using ultra-high-performance liquid chromatography coupled with high-resolution tandem mass spectrometry (UHPLC–HRMS/MS) [36,37,38]. Chromatographic separation was conducted on a Vanquish Horizon UHPLC system (Thermo Fisher Scientific, Waltham, MA, USA) equipped with an Accucore Phenyl-Hexyl analytical column (100 mm × 2.1 mm, 2.6 μm particle size). The mobile phase consisted of water containing 0.1% formic acid (solvent A) and acetonitrile containing 0.1% formic acid (solvent B). Elution was performed using a gradient program initiated at 5% solvent B, gradually increased to 95% solvent B over 18 min, maintained for 4 min, and subsequently re-equilibrated to initial conditions. The flow rate was maintained at 0.30 mL/min, and the injection volume was 5 μL.

Mass spectrometric detection was carried out using an Orbitrap Exploris 240 mass spectrometer (Thermo Fisher Scientific) equipped with a heated electrospray ionization source operating in both positive and negative ionization modes. Full MS scans were acquired over an *m*/*z* range of 70–1500 with a resolving power of 60,000 FWHM. Data-dependent MS/MS spectra were obtained for structural elucidation of detected metabolites. Metabolite annotation was performed using Compound Discoverer 3.3 software with integrated searches against mzCloud, ChemSpider, HMDB, METLIN, and LipidMaps databases. Metabolites were considered putatively identified when mass accuracy was below 5 ppm and fragmentation patterns matched reference spectra.

To ensure analytical robustness, pooled quality-control (QC) samples were generated by combining equal aliquots from all extracts and injected every six analytical runs. Analytical reproducibility was evaluated through principal component analysis and coefficient of variation (CV) calculations. Only metabolite features exhibiting CV values below 30% across QC replicates were retained for downstream analyses.

### 4.3. Identification of Osteosarcoma-Associated Genes and Ferroptosis-Related Targets

Transcriptomic analysis was performed using the publicly available osteosarcoma dataset GSE42352 obtained from the Gene Expression Omnibus (GEO) database [39,40]. Raw expression matrices were downloaded and processed in R software (version 4.4.0). Data normalization and differential expression analyses were conducted using the Limma package. Genes exhibiting |log2 fold change| > 1 and adjusted *p*-values < 0.05 were considered significantly dysregulated.

To specifically investigate ferroptosis-associated molecular signatures and bone tumor microenvironment remodeling, differentially expressed genes were integrated with curated gene sets obtained from GeneCards, DisGeNET, OMIM, FerrDb, CTD, and MalaCards databases. Osteosarcoma-associated genes, ferroptosis-related genes, and bone remodeling genes were merged and deduplicated prior to subsequent analyses. Candidate hub genes were prioritized based on fold-change magnitude, biological relevance, and network topology parameters.

### 4.4. Compound Target Prediction and Network Pharmacology Analysis

The chemical structures of identified *H. scabra* metabolites were retrieved from PubChem and converted into canonical SMILES format [41]. Potential protein targets were predicted using SwissTargetPrediction, SEA Search Server, BindingDB, STITCH, and ChEMBL databases. Predicted targets from all compounds were merged to generate a comprehensive metabolite-target dataset.

Intersecting targets shared among *H. scabra* metabolites, osteosarcoma-associated genes, and ferroptosis-related genes were identified using Venny 2.1 and visualized as Venn diagrams. Protein–protein interaction (PPI) analysis was subsequently performed using STRING database version 12.0 with a confidence score threshold of 0.700. Network topology analysis was conducted using Cytoscape version 3.10.1 to identify central hub genes according to degree centrality, betweenness centrality, and closeness centrality metrics.

### 4.5. Functional Enrichment Analysis

Biological functions and signaling pathways associated with overlapping targets were investigated using Gene Ontology (GO) and Kyoto Encyclopedia of Genes and Genomes (KEGG) enrichment analyses [42]. Enrichment analyses were conducted using the clusterProfiler package in R. Significantly enriched biological processes, molecular functions, cellular components, and signaling pathways were identified using a false discovery rate (FDR) threshold of <0.05. Visualization of enrichment results was performed using clusterProfiler, enrichplot, ggplot2, and EnhancedVolcano packages. Pathways related to ferroptosis, HIF-1 signaling, MAPK signaling, focal adhesion, cellular senescence, angiogenesis, and osteoclastogenesis were prioritized for interpretation.

### 4.6. Molecular Docking Analysis

Molecular docking simulations were performed to evaluate interactions between major *H. scabra* metabolites and key osteosarcoma-associated proteins, including CXCR4 (PDB ID: 3ODU), CTSK (PDB ID: 5TUN), RUNX2 (PDB ID: 6VGG), VEGFA (PDB ID: 1FLT), and TFRC (PDB ID: 1CX8). Three-dimensional protein structures were downloaded from the RCSB Protein Data Bank, while ligand structures were obtained from PubChem.

Docking analyses were conducted using CB-Dock3, which integrates cavity detection with AutoDock Vina version 1.2.3 scoring [43]. The top-ranked binding cavities automatically identified by CB-Dock3 were selected for docking calculations. Docking exhaustiveness was set to 12, while cavity dimensions and center coordinates were automatically optimized according to predicted active-site geometry. Binding affinity values were expressed as kcal/mol, with more negative values indicating stronger ligand–protein interactions. Doxorubicin, methotrexate, and erastin were included as reference compounds.

### 4.7. Cell Culture and Treatment

Human osteosarcoma MG-63 cells (ATCC^®^ CRL-1427™) and murine macrophage RAW264.7 cells (ATCC^®^ TIB-71™) were obtained from the American Type Culture Collection (ATCC, Manassas, VA, USA) [44]. MG-63 cells were maintained in Dulbecco’s Modified Eagle Medium (Thermo Fisher Scientific, Grand Island, United States) supplemented with 10% fetal bovine serum and 1% penicillin–streptomycin. RAW264.7 cells were cultured under identical conditions. Cells were maintained at 37 °C in a humidified atmosphere containing 5% CO_2_.

For experimental treatments, cells were exposed to *H. scabra* extract at concentrations of 25, 50, and 100 μg/mL for 72 h. Erastin (10 μM) served as a ferroptosis-positive control. Vehicle-treated cells receiving 0.1% DMSO served as controls.

### 4.8. Cell Viability Assay

Cell viability was determined using the MTT assay [45]. MG-63 cells were seeded in 96-well plates at a density of 1 × 10^4^ cells/well and allowed to attach overnight. Following treatment, 20 μL of MTT solution (5 mg/mL) was added to each well and incubated for 4 h. Formazan crystals were dissolved using DMSO, and absorbance was measured at 570 nm using a microplate reader. Cell viability was expressed as a percentage relative to untreated controls.

### 4.9. Wound-Healing Migration Assay

MG-63 cells were seeded in six-well plates and cultured until approximately 90% confluence [46]. A sterile 200 μL pipette tip was used to generate a uniform scratch across the monolayer. Detached cells were removed by PBS washing before treatment. Images were captured immediately (0 h) and after 24 and 48 h using an inverted microscope. Migration was quantified as percentage wound closure using ImageJ software version 1.53.

### 4.10. Evaluation of Ferroptosis-Associated Biomarkers

Following treatment, expression levels of GPX4, NRF2, and TFRC were quantified using quantitative real-time PCR. Total RNA was isolated using TRIzol reagent, reverse-transcribed into cDNA, and amplified using SYBR Green chemistry. Relative gene expression was calculated using the 2^−ΔΔCt^ method with GAPDH as the housekeeping gene.

Intracellular malondialdehyde (MDA) concentrations were quantified using a lipid peroxidation assay kit, whereas reduced glutathione (GSH) levels were measured using a commercial colorimetric assay kit according to manufacturers’ instructions. All measurements were performed in triplicate.

### 4.11. MG-63/RAW264.7 Co-Culture Model and Osteoclastogenesis Assay

To mimic the bone tumor microenvironment, MG-63 cells were co-cultured with RAW264.7 osteoclast precursor cells using Transwell inserts [47,48]. MG-63 cells were seeded into the lower chambers of six-well plates at a density of 2 × 10^5^ cells/well and allowed to adhere overnight. RAW264.7 cells were seeded into Transwell inserts (0.4 μm pore size) at a density of 1 × 10^5^ cells/insert. The pore size permitted the exchange of soluble factors between cell populations while preventing direct cell–cell contact.

Following cell attachment, the co-culture system was maintained in complete DMEM supplemented with 10% fetal bovine serum and 1% penicillin–streptomycin. Osteoclast differentiation of RAW264.7 cells was induced by treatment with recombinant mouse RANKL (50 ng/mL) for 7 days, with medium replacement every 2–3 days. Simultaneously, co-cultures were treated with *Holothuria scabra* extract at concentrations of 25, 50, and 100 μg/mL. Vehicle-treated co-cultures containing 0.1% DMSO served as controls.

At the end of the incubation period, protein concentrations of RANKL, VEGFA, and MMP9 in the MG-63/RAW264.7 co-culture supernatants were quantified using commercially available ELISA kits according to the manufacturers’ instructions. Osteoclast differentiation was assessed by tartrate-resistant acid phosphatase (TRAP) staining using a commercial TRAP staining kit according to the manufacturer’s instructions. Following 7 days of RANKL-induced differentiation and treatment with *H. scabra* extract, RAW264.7 cells were washed twice with phosphate-buffered saline (PBS) and fixed with 4% paraformaldehyde for 15 min at room temperature. Fixed cells were subsequently incubated with TRAP staining solution containing naphthol AS-BI phosphate substrate and Fast Red Violet LB salt under protected light conditions until positive staining became visible.

TRAP-positive cells exhibiting a distinct red-purple coloration and containing three or more nuclei were identified as mature osteoclasts. Stained cells were examined under a light microscope at 200× magnification. Osteoclast formation was quantified by counting TRAP-positive multinucleated cells in five randomly selected microscopic fields per well. The average number of TRAP-positive osteoclasts per field was calculated and used for statistical analysis.

### 4.12. Statistical Analysis

All experiments were performed using three independent biological replicates and expressed as mean ± standard deviation (SD). Comparisons among multiple groups were performed using one-way analysis of variance (ANOVA) followed by Tukey’s post hoc test. Differences were considered statistically significant at *p* < 0.05.

## 5. Conclusions

This study provides the first comprehensive multi-omics-guided evaluation of the anti-osteosarcoma potential of *H. scabra* metabolites through the integration of metabolomic profiling, transcriptomic analysis, network pharmacology, molecular docking, and biological validation. LC–MS/MS analysis identified triterpene glycosides and sterol compounds, including holothurins, scabrasides, fucosterol, and desmosterol, as major bioactive constituents with promising anticancer properties. Integrated multi-omics analyses revealed key therapeutic targets associated with ferroptosis and bone tumor microenvironment remodeling, including CXCR4, CTSK, RUNX2, VEGFA, and TFRC. Molecular docking demonstrated strong multi-target interactions of several metabolites, particularly holothurin A and fucosterol, with these osteosarcoma-associated proteins. Functional validation confirmed that *H. scabra* extract significantly inhibited MG-63 cell viability and migration, induced a ferroptosis-associated molecular phenotype characterized by suppression of the GPX4–NRF2–GSH axis and elevation of TFRC and lipid peroxidation, and attenuated osteoclastogenesis, angiogenesis, and extracellular matrix remodeling in a MG-63/RAW264.7 co-culture model. Collectively, these findings suggest that *H. scabra* exerts anti-osteosarcoma activity through a dual mechanism involving ferroptosis induction and suppression of the bone tumor microenvironment. This study highlights *H. scabra* as a promising marine source of multi-target therapeutic candidates and provides a mechanistic foundation for future in vivo and translational investigations aimed at developing novel marine-derived interventions for osteosarcoma management. By simultaneously targeting tumor-intrinsic ferroptotic vulnerabilities and tumor-supportive microenvironmental signaling, *H. scabra* metabolites represent promising next-generation marine-derived therapeutics for osteosarcoma and potentially other bone-associated malignancies.

## Figures and Tables

**Table 1:** Putatively Identified Metabolites from *Holothuria scabra* Extract Based on LC–MS/MS Profiling.

Compound	Compound Class	Molecular Formula	Exact Mass	Precursor *m*/*z*	Adduct	Retention Time (min)	Peak Area	Relative Abundance (%)
Holothurin A	Saponin	C_54_H_86_O_28_	1190.24	1213.23	[M + Na]^+^	18.54	856,000,000	12.8
Holothurin B	Saponin	C_54_H_84_O_27_	1172.22	1195.21	[M + Na]^+^	17.98	812,000,000	12.1
Scabraside A	Saponin	C_56_H_88_O_29_	1234.27	1257.26	[M + Na]^+^	17.83	792,000,000	11.8
Scabraside D	Saponin	C_54_H_84_O_27_	1172.22	1195.21	[M + Na]^+^	16.35	625,000,000	9.3
24-Methylenecholesterol	Sterol	C_28_H_46_O	398.66	399.67	[M + H]^+^	23.82	298,000,000	4.5
Fucosterol	Sterol	C_29_H_48_O	412.69	413.7	[M + H]^+^	24.31	367,000,000	5.5
Desmosterol	Sterol	C_27_H_44_O	384.64	385.65	[M + H]^+^	22.11	201,000,000	3
Arachidonic Acid	Fatty Acid	C_20_H_32_O_2_	304.47	305.48	[M + H]^+^	12.31	146,000,000	2.2
Fahrunicoline Nicolasine	Pentapeptide	C_30_H_47_N_9_O_10_	693.34	716.33	[M + Na]^+^	7.45	1.42 × 10^6^	1.24

**Table 2:** Candidate Hub Genes Associated with Ferroptosis and Bone Tumor Microenvironment Remodeling, Identified from the GSE42352 Osteosarcoma Dataset.

Rank	Gene Symbol	Gene Name	logFC	AveExpr	*p*.Value	adj.*p*.Value	B	Regulation	Functional Category
1	*VEGFA*	Vascular endothelial growth factor A	2.54	8.12	1.2 × 10^−8^	3.4 × 10^−6^	12.5	Up	Angiogenesis
2	*MMP9*	Matrix metalloproteinase 9	2.31	7.88	2.8 × 10^−8^	5.1 × 10^−6^	11.8	Up	Invasion
3	*CXCR4*	C-X-C motif chemokine receptor 4	2.08	7.41	6.5 × 10^−8^	8.4 × 10^−6^	11.1	Up	Metastasis
4	*IL6*	Interleukin 6	1.97	6.92	1.1 × 10^−7^	0.000013	10.7	Up	Inflammation
5	*HMOX1*	Heme oxygenase 1	1.86	8.01	2.4 × 10^−7^	0.00002	10.1	Up	Ferroptosis
6	*TFRC*	Transferrin receptor	1.74	7.55	3.2 × 10^−7^	0.000027	9.8	Up	Ferroptosis
7	*ACSL4*	Acyl-CoA synthetase long-chain family member 4	1.63	7.12	4.7 × 10^−7^	0.000032	9.5	Up	Ferroptosis
8	*TGFB1*	Transforming growth factor beta 1	1.58	7.33	6.1 × 10^−7^	0.000041	9.2	Up	Bone microenvironment
9	*TNFSF11*	RANKL	1.49	6.81	8.5 × 10^−7^	0.00005	8.9	Up	Osteoclastogenesis
10	*CTSK*	Cathepsin K	1.41	6.44	1.1 × 10^−6^	0.000062	8.5	Up	Osteoclastogenesis
11	*GPX4*	Glutathione peroxidase 4	−1.32	8.2	2.5 × 10^−6^	0.00011	7.8	Down	Ferroptosis
12	*SLC7A11*	Solute carrier family 7 member 11	−1.21	7.6	3.4 × 10^−6^	0.00015	7.3	Down	Ferroptosis
13	*FTH1*	Ferritin heavy chain 1	−1.18	8.45	0.000005	0.0002	6.9	Down	Iron metabolism
14	*TNFRSF11B*	OPG	−1.12	6.52	7.4 × 10^−6^	0.00025	6.5	Down	Bone remodeling
15	*RUNX2*	RUNX family transcription factor 2	−1.05	7.21	0.000009	0.0003	6.1	Down	Osteoblast differentiation

**Table 3:** PASS Prediction, Toxicological Assessment, and Drug-Likeness Evaluation of Major *Holothuria scabra* Metabolites.

Compounds	Pa Score			Toxicity Model Computation Analysis		Drug-Likeness		
	Chemopreventive	Antiproliferative Potential	Myc Inhibitor	Predicted LD_50_ (mg/kg)	Toxicity Class	Lipinski Rule	Pfizer Rule	GSK
Holothurin A	NA			3220	5	Rejected	Accepted	Rejected
Holothurin B	NA			3220	5	Rejected	Accepted	Rejected
Scabraside A	NA			4000	5	Rejected	Accepted	Rejected
Scabraside D	0.856	0.746	0.433	2190	5	Rejected	Accepted	Rejected
24-Methylenecholesterol	0.818	0.795	0.753	890	4	Accepted	Rejected	Rejected
Fucosterol	0.809	0.763	0.748	890	4	Accepted	Rejected	Rejected
Desmosterol	0.891	0.842	0.754	890	4	Accepted	Rejected	Rejected
Arachidonic Acid	0.335	0.519	0.462	10,000	6	Accepted	Rejected	Rejected
Fahrunicoline Nicolasine	NA	NA	NA	2400	5	Rejected	Accepted	Rejected

NA: Not Available; Molecular Charge = −1.

**Table 4:** Molecular Docking Scores of *Holothuria scabra*-Derived Metabolites against Key Osteosarcoma-Related Targets.

Compounds	Core Protein Related to Osteosarcoma				
	CXCR4 (PDB ID: 3ODU)	CTSK (PDB ID: 5TUN)	RUNX2 (PDB ID: 6VGG)	VEGFA (PDB ID: 1FLT)	TFRC (PDB ID: 1CX8)
Doxorubicin (Control)	−10.3	−6.9	−8.1	−7.9	−9.0
Methotrexate (Control)	−8.4	−7.5	−8.2	−8.3	−10.4
Erastin (Control)	−9.8	−7.1	−9.6	−7.4	−9.5
Holothurin A	−10.6	−9.0	−9.5	−8.8	−11.0
Holothurin B	−10.2	−7.1	−8.6	−7.4	−10.2
Scabraside A	−10.4	−7.3	−9.5	−8.5	−10.7
Scabraside D	−10.1	−7.2	−8.5	−7.7	−9.7
24-Methylenecholesterol	−9.6	−6.6	−8.6	−7.0	−9.7
Fucosterol	−11.4	−6.5	−9.0	−7.4	−9.7
Desmosterol	−9.9	−6.1	−8.2	−7.5	−10.3
Arachidonic Acid	−6.6	−5.4	−6.7	−5.8	−7.5
Fahrunicoline Nicolasine	−8.2	−6.3	−7.2	−7.4	−8.6

## Data Availability

The original contributions presented in this study are included in the article. The datasets used and/or analysed during the current study are available from the corresponding authors on reasonable request. Further inquiries can be directed to the corresponding authors.
